# 

**Published:** 1892-12-03

**Authors:** 


**Notices of Births, Deaths, and Marriages,** are inserted in this column at a charge of 2s. 6d. for an announcement not exceeding four lines.

### NOTICE TO CORRESPONDENTS.

All MS., Letters, Books for Review, and other matters intended for the Editor should be addressed The Editor, The Lodge, Porchester Square, London, W.

The Editor cannot undertake to return rejected M.S., even when accompanied by stamped directed envelope.

**Notice to Advertisers and Subscribers.**

All Advertisements, Orders for Copies of The Hospital, and Business Communications should be addressed to The Publisher, *not to the Editor*, The Hospital, 140, Strand, W.C.

The Cost of Subscription, post free, is as follows:—Per week, 2½d.; per quarter, 2s. 6d.; per half-year, 5s.; per annum, 8s. 6d. Foreign:—Per annum, 10s. 8d.; payable, in all cases, in advance.

**"Burdett's Hospital Annual," 1892—1893.**

Fourth year of publication. 700 pp. Boards, gilt lettering. A most complete guide to British and Colonial Hospitals, Dispensaries, Nursing and Convalescent Institutions, and Asylums. Price 5s. Published by The Scientific Press (Limited), 140, Strand, W.C.

**NOTICE.—The Index for Vol. XII. (ending September, 1892) is on sale at the Office of this Journal, price 2½d., post free.**

### Books Received.

Whittaker and Co.

"Dissections Illustrated." By C. Gordon Brodie, F.R.C.S.

J. and A. Churchill

"Reform in the Treatment of the Insane." By D. Hack Tuke, M.D.

"The Value of Hypnotism and Chronic Alcoholism." By Chas. L. Tuckey, M.D.

Fisher Unwin.

"The Nationalisation of Health." By Havelock Ellis.

K. Lewis and Co.

"History of Medical Education." By Dr. Puschmann; translated by Evan H. Hare.

### THE STUDENT OF MEDICINE.

[All communications for this Supplement should be addressed to THE EDITOR of THE HOSPITAL, at 140, Strand, with the words, "Students' Column," written in the left hand top corner of the envelope.]

### NOTES FROM THE SCHOOLS.

### Westminster Hospital. Guthrie Society.

The question discussed by this society at the last monthly meeting, on Thursday, was, "Is Vivisection Justifiable?" Mr. Wolseley-Lewis opened the debate by reading a short paper, in which he upheld that vivisection was justifiable. Mr. Walter Spencer, Dr. Donkin, Mr. Nelson, and two or three other speakers also took this view; while Messrs. Nitch-Smith and Winckworth and the chairman (Dr. Hebb) endeavoured strongly to uphold the opposite view. Eventually the following proposal was put before the society by Mr. Winckworth: "That the past history of vivisection cannot be considered satisfactory, and that in the opinion of this society the strictest legislation is necessary to prevent cruelty being done to animals under the cover of science." On counting the votes the proposal was rejected by a large majority.

### Edinburgh University.

The following have been elected as representatives for the Faculty of Medicine on the Students' Representative Council:—First year: J. L. Chiene, J. G. Rosse, J. Prentice, and G. L. Milne. Second year: W. E. Gibbons, G. Henderson, H. Casdale, A. Steel, C. M'Cammon, E. Arkwright, J. O. Cuthbertson, and D. Waterston. Third year: H. H. Balfour, J. R. Muir, J. A. Laing, G. F. H. Cunningham, J. D. C. Howden, H. J. F. Simson, D. Hutton, and H. O. Hobson. Fourth year: W. J. Garbutt, R. W. Briggs, R. H. Makgill, T. A. Glover, C. J. R. McFadden, G. H. Ruston Harrison, and A. P. Steavenson. Fifth year: A. A. Scot-Skirving, J. G. Cattanach, A. M. Easterbrook, S. J. Aarons, O. H. Isard, E. M. Skeete, G. St. Clair Thorn, and R. Hutchison.

---

### PASS LISTS.

### University of London.

M.B. EXAMINATION: OCTOBER, 1892.

FIRST DIVISION.—C. Addison, St. Bartholomew's Hospital; Louisa B. Aldrich-Blake, London School of Medicine and Royal Free Hospital; H. A. Ballance, University College; C. Barker, Sheffield Medical Institution and St. Bartholomew's Hospital; A. S. Blackwell (B.Sc.), St. Bartholomew's Hospital; W. Bligh, Guy's Hospital; C. R. Box (B.Sc.), St. Thomas's Hospital; A. E. Brindley, Owen's College and Manchester Royal Infirmary; T. G. Brodie, King's College; F. J. Coleman, Guy's Hospital; A. M. Daldy, Guy's Hospital; C. C. Elliott, Guy's Hospital; J. Evans, University Colleges of Liverpool and London; S. G. Floyd, Guy's Hospital; E. T. E. Hamilton (B.Sc.), Guy's Hospital; R. Hamilton, Owen's College and Manchester Royal Infirmary; Jessie F. Hatch, London School of Medicine for Women; A. D. Heath, University College; G. S. Johnston, Queen's College, Birmingham; J. Jones, University College; W. B. Jones, St. Bartholomew's Hospital; V. W. Low, St. Mary's Hospital, C. B. T. Musgrave, University College; C. S. Pantin, Guy's Hospital; H. T. Parker, St. Bartholomew's Hospital; S. H. Perry, Queen's College, Birmingham; W. L. Pethybridge (B.Sc.), St. Bartholomew's Hospital; W. J. Procter, London Hospital and University College; W. P. Purvis (B.Sc.), St. Thomas's Hospital; M. Randall (B.A.), University College; F. A. Roberts, the Yorkshire College; L. Rogers, St. Mary's Hospital; C. M. Rogerson, Yorkshire College and General Infirmary, Leeds; Mary E, kye, London School of Medicine and Royal Free Hospital; H. S. Sandifer, King's College; E. W. Selby, University College; A. J. Sharp, Guy's Hospital; H. R. Smith, University College; C. G. Spencer, University College; G. A. Stephens (B.Sc.), University College; T. R. Taylor (B.Sc), Guy's Hospital; T. M. Tibbetts, Queen's College, Birmingham; S. G. Toller, St. Thomas's Hospital; F. W. Wesley, University College; C. E. Wheeler (B.Sc.), St. Bartholomew's Hospital; and A. Whitfield, King's College.

SECOND DIVISION.—H. S. Ballance, King's College; R. H. Cole, St. Mary's Hospital; H. Distin, King's College; H. W. Gibson, Middlesex Hospital; C. J. Harrison, University College; T. D. Lister, Guy's Hospital; J. W. Pugh, London Hospital; H. Ramsden, Owen's College; H. E. Tracey, St. Bartholomew's Hospital; and S. L. B. Wilks, the Yorkshire College.

---

### VACANCIES.

### Resident Medical Officers.

Birmingham and Midland Eye Hospital, Church Street, Birmingham, Assistant House Surgeon—Salary £50 per annum, with apartments and board. Applications to the Chairman of the Medical Board by December 8th.

Brighton, Hove, and Preston Dispensary, House Surgeon to the Western Branch, unmarried, doubly qualified—Salary £140 per annum (less board at 8s. per week), with furnished apartments, coal, gas, and attendance. Applications to the Assistant Secretary by December 15th.

### THE STUDENT OF MEDICINE—(*continued*).

City of London Hospital for Diseases of the Chest, Victoria Park, E., House Physician—Board, residence, and allowance for washing provided. Appointment for six months. Applications to the Secretary at the Office, 24, Finsbury Circus, E.C., by December 8th.

Farringdon General Dispensary, Bartlett's Buildings, Holborn Circus, E.C., Resident Medical Officer—Salary £100 per annum, with apartments and attendance. Applications to W. K. Taunton, Honorary Secretary, by December 5th.

Noble's Isle of Man General Hospital and Dispensary, Athol Street, Douglas, Isle of Man, Resident House Surgeon, doubly qualified, unmarried—Salary, £100 per annum, with apartments, gas, coal, and laundry free. Applications to F. Brown, Honorary Secretary, by December 3rd.

North-Eastern Hospital for Children, Hackney Road, N.E., Junior House Surgeon, doubly qualified—Salary £60 per annum. Applications to Alfred Nixon, Secretary, 27, Clement's Lane, E.C., by December 9th.

Northumberland County Lunatic Asylum, Morpeth, Assistant Medical Officer, unmarried—Salary £120 per annum, increasing £10 yearly to £150, with furnished apartments, and board and lodging. Applications to Dr. McDowall at the Asylum by December 15th.

Royal Infirmary of Edinburgh, Superintendent, must be member of medical profession—Salary £500 per annum, with free house and gas. Applications to Mr. James S. Trainer, Treasurer and Clerk, by December 5th.

West London Hospital, Hammersmith Road, W., House Physician. Appointment for six months. Board and lodging provided. Applications to R. J. Gilbert, Secretary, by December 7th.

West London Hospital, Hammersmith Road, W., House Surgeon. Appointment for six months. Board and lodging provided. Applications to R. J. Gilbert, Secretary, by December 7th.

Westminster General Dispensary, 9, Gerrard Street, Soho, Resident Medical Officer. Appointment for one year. Applications to J. J. Johnson, Secretary, by December 15th.

### Non-Resident Medical Officers.

Metropolitan Hospital, Kingsland Road, N.E., Assistant Physician. Applications to Charles H. Byers, Secretary, by November 28th.

Samaritan Hospital for Diseases of Women, Lisburn Road, Belfast, Two Honorary Attending Physicians. Applications to the Secretaries by December 5th.

Victoria Hospital, Burnley, Honorary Ophthalmic and Aural Surgeon. Applications, endorsed "Ophthalmic," to the Honorary Secretary, Joshua Rawlinson, J.P., by December 6th.

